# Efficacy and Safety of Oral Isotretinoin in Plane Warts: A Systematic Review on Clinical Studies

**DOI:** 10.1155/sci5/4242268

**Published:** 2025-10-22

**Authors:** Bahareh Abtahi-Naeini, Hossein Sattari, Fereshte Rastegarnasab, Sarah Seyedyousefi

**Affiliations:** ^1^Skin Diseases and Leishmaniasis Research Center, Isfahan University of Medical Sciences, Isfahan, Iran; ^2^Pediatric Dermatology Division of Department of Pediatrics, Imam Hossein Children's Hospital, Isfahan University of Medical Sciences, Isfahan, Iran; ^3^Clinical Research Development Center, Najafabad Branch, Islamic Azad University, Najafabad, Iran; ^4^Student Research Committee, Isfahan University of Medical Sciences, Isfahan, Iran; ^5^Hypertension Research Center, Cardiovascular Research Institute, Isfahan University of Medical Sciences, Isfahan, Iran

**Keywords:** dermatology, isotretinoin, plane wart, systematic review, viral wart

## Abstract

**Background:**

Human papillomavirus–induced plane warts represent benign cutaneous lesions that, despite frequent spontaneous resolution, demonstrate persistence and recurrence patterns associated with considerable cosmetic and psychological morbidity. Isotretinoin has emerged as a therapeutic consideration for recalcitrant plane warts. We evaluated isotretinoin effectiveness and safety in the management of plane warts through a systematic analysis of available clinical evidence.

**Methods:**

We conducted this systematic review following Preferred Reporting Items for Systematic Review and Meta-Analysis guidelines, with prospective registration in PROSPERO (ID: CRD420251060689). Comprehensive searches of PubMed, Scopus, and EMBASE databases were extended through November 2024. Primary efficacy outcomes focused on isotretinoin-induced changes in wart lesion characteristics.

**Results:**

Fifteen studies met the inclusion criteria, comprising six clinical trials, one cross-sectional study, six case reports, and two case series conducted across seven countries, encompassing 438 patients. Complete resolution rates demonstrated substantial variation, ranging from 38% to 87.5%. Among 10 studies documenting adverse events, cheilitis and dry skin emerged as the most common side effects.

**Conclusion:**

Oral isotretinoin shows promise for recalcitrant plane warts, but current evidence is heterogeneous and largely of moderate-to-low quality. These findings are insufficient to support routine clinical use at this time; well-designed, adequately powered randomized controlled trials with standardized dosing and outcomes are needed.

## 1. Introduction

Plane warts are benign skin lesions caused by human papillomavirus (HPV), predominantly types 3, 10, and 28. They commonly appear as flat-topped, slightly raised, frequently multiple, skin-colored papules often involving the face and hands among children and young adults [1, 2]. While they often resolve spontaneously, persistence and recurrence may occur and can lead to significant cosmetic and psychological concerns [3].

Current treatment options vary based on the number, location, and persistence of lesions, including topical agents such as salicylic acid and retinoids, cryotherapy, laser therapy, immunomodulatory agents, and surgery [1, 4, 5]. Laser therapy is often preferred for recalcitrant or extensive warts, as it offers high precision, minimal scarring, and reduced recurrence rates [6].

Topical isotretinoin is reported to be used for the treatment of dermatological conditions such as mild-to-moderate acne vulgaris, acne scarring, post-inflammatory hyperpigmentation, freckling, solar lentigines, fine wrinkling, solar comedone, sun-induced skin fragility, actinic keratoses, melasma, oral lichen planus, and Darier disease. Topical isotretinoin offers a targeted approach with fewer systemic side effects [7–11].

Oral isotretinoin was approved by the FDA in 1982 for the treatment of severe recalcitrant nodular acne that has not responded to conventional therapies. However, it has been off-label used for the treatment of mild-to-moderate acne, cutaneous T-cell lymphomas, neuroblastoma, rosacea, folliculitis, facial pyoderma, and the prevention of squamous cell carcinoma in high-risk patients [12, 13].

The concept of employing oral isotretinoin as a therapeutic strategy for plane warts was first introduced in a 2008 case report by Rallis et al. [14]. This report described a 20-year-old male soldier exhibiting flat-to-papillomatous wart-like lesions on his face, which were similar to plane warts presented in his early childhood and proved resistant to topical retinoids and cryotherapy. The patient received oral isotretinoin at a dosage of 0.8 mg/kg/day for approximately 6 months, achieving complete resolution of the lesions 1 month prior to the treatment's completion. This initial finding prompted further exploration, notably through studies by Al-hammy et al. and Miljkovic et al. in 2012 [15, 16], followed by 12 additional investigations in subsequent years up to the present.

Despite multiple destructive and topical options, responses are variable and relapse is common, particularly when lesions koebnerize or are multifocal, underscoring an unmet need for effective systemic approaches. Isotretinoin is mechanistically attractive in plane warts because retinoids modulate keratinocyte proliferation and differentiation and may suppress HPV DNA replication, with additional immunomodulatory and antiangiogenic effects that could limit persistence and spread. These properties provide a biologic rationale to evaluate oral isotretinoin for recalcitrant or widespread disease [17–19].

Despite emerging evidence suggesting a role for isotretinoin for managing plane warts, no comprehensive systematic review has yet been synthesized to assess its efficacy and safety. Therefore, this systematic review aims to evaluate the effectiveness and safety of isotretinoin in the treatment of plane warts by considering existing clinical studies to assess its potential role as a therapeutic option.

## 2. Methods

### 2.1. Search Strategy

This review was conducted according to the Preferred Reporting Items for Systematic Reviews and Meta-analyses (PRISMA) guidelines [20] and has been registered in PROSPERO. Also, the protocol for this study was approved by the Sciences and Ethics Committee of Isfahan University of Medical Sciences. We tried to retrieve manuscripts related to isotretinoin and its effect on plane warts. PubMed, Scopus, and EMBASE were thoroughly searched up to November 2024.

To ensure a comprehensive and pertinent search, a combination of Medical Subject Headings (MeSH) terms and free-text keywords was utilized. We utilized a systematic review approach using the following keywords: “isotretinoin,” “13-cis-retinoic acid,” “Roaccutane,” “accutane,” “absorica,” “myorisan,” “claravis,” “amnesteem,” “isotretinoin zinc salt,” “ro 4 3780,” “ro 43780,” “isotretinoinum,” “wart,” “verruca,” “verrucae,” “plane,” “plana,” “planae,” “flat,” “juvenile,” “planar,” “verruca plana,” “verrucae planae.” These keywords were combined using the Boolean operators “OR” and “AND.” We also searched by hand and scanned previous manuscripts' reference lists to identify other related studies. A complete search strategy can be found in the Supporting Information file (available here).

### 2.2. Selection Criteria and Data Extraction

Clinical studies that reported using isotretinoin for the treatment of plane warts in humans, including randomized controlled trials (RCT), quasiexperimental studies, observational studies, case reports, and case series, were considered eligible for inclusion in this review. Studies with insufficient data, studies in languages other than English, conference papers, review articles, in vitro studies, pharmacological (pharmacokinetics) studies, and unpublished studies were considered ineligible to be included. The primary outcome of interest was the treatment efficacy of oral isotretinoin, classified as a change in wart lesions where possible.

During the selection phase, Zotero software was employed to manage and organize references throughout the preparation and submission of the systematic review manuscript on isotretinoin. Following the removal of duplicates, two researchers (F.R. and S.S.) independently assessed the articles based on their titles and abstracts. Disagreements were resolved by consulting a third researcher (H.S.) for a final decision. In the next step, the full text of the eligible studies was retrieved and assessed. Efforts were made to secure the full text of all included articles, including contacting corresponding authors when necessary. Furthermore, the reference lists of the included papers were manually reviewed to ensure that no relevant publications were missed in the database searches.

Two independent reviewers assessed the quality of the final manuscripts, after which data extraction was performed. The data collection process included various general characteristics of each study, such as the author's name, publication year, and study design. It also involved specific details about the study population, including sample size categorized by sex and the ages of participants. Furthermore, information regarding the medical condition under treatment, the intervention or dosage and duration of treatment, the primary outcome, the site of lesions, and any adverse events was systematically gathered.

### 2.3. Synthesis Methods

Given substantial methodological heterogeneity—mixed study designs (RCTs, quasiexperimental, cross-sectional, case series/reports), variable dosing strategies (fixed vs. weight-based; 0.1–0.8 mg/kg/day), heterogeneous comparators, treatment durations (1–12 months), and nonstandardized outcome definitions (e.g., “complete clearance,” “partial response,” “improvement”)—a quantitative meta-analysis was not appropriate. Many studies lacked control groups and did not provide variance estimates compatible with pooling. We therefore conducted a structured narrative synthesis, grouping studies by design and dose regimen and qualitatively summarizing efficacy and safety signals.

## 3. Quality Assessment of Included Articles

The Joanna Briggs Institute (JBI) Critical Appraisal Checklists [21] were used to assess the methodological quality of the included studies. These instruments provide tailored criteria for various study designs, encompassing case reports, case series, observational studies, and clinical trials. It is noteworthy that all studies were subjected to data extraction and analysis, irrespective of their methodological quality ratings. This methodology ensures a thorough examination of the entire body of available evidence. The detailed outcomes of these critical appraisals are documented in five separate tables within the Supporting Information, ensuring transparency and enabling readers to evaluate the quality of the included studies.

## 4. Results

**Figure 1:**
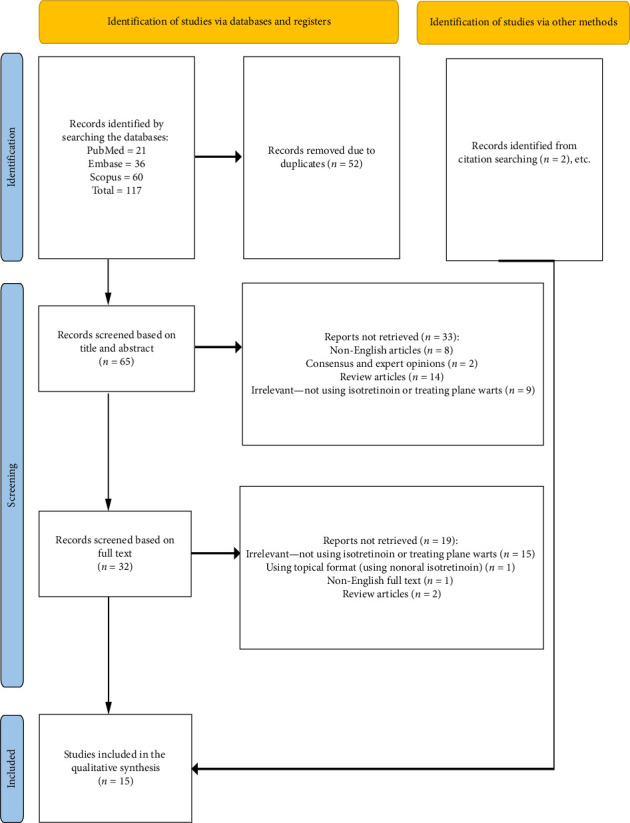
PRISMA flow diagram for included studies.

Fifteen studies were eventually included in this review. These studies included 6 clinical trials [10, 16, 22–25], 1 cross-sectional study [26], 6 case reports [1, 2, 14, 27–29], and 2 case series [15, 30]. Most of the rigorous evidence comes from clinical trials (6/15 studies). Studies were conducted across 7 countries. Out of our 15 studies, 4 studies took place in India [10, 26, 29, 30], 2 in Iraq [16, 22], 2 in Egypt [24, 25], 2 in Mexico [2, 23], and 1 for each of Greece [14], Slovenia [15], Poland [1], Turkey [28], and the USA [27]. All the participants involved was 438 patients with the most of them were from Egypt (208 patients), followed by India (126), Iraq (66), and Mexico (27). The largest study with the most patients included 108 patients diagnosed with warts.  Figure 1 depicts the flowchart of the included studies, and the details and characteristics of the included studies are summarized in Table 1.

In retrieved studies, sample sizes varied greatly, from single case reports to clinical trials with up to 108 patients. Larger studies generally had more reliable but variable resolution rates from 44.4% to 87% [24–26]. Female patients outnumbered male patients in most studies that reported gender distribution. Ages ranged widely from children to adults (youngest was 13 years, oldest mean age group was 31.56 years), while the treatment appears effective across age groups. In terms of lesion location, face was the most common site which was mentioned in 6 studies [2, 10, 14–16, 28]. Some studies included generalized warts (3 studies) [27, 29, 30] or extremities.

About the drug dosing used in the studies, the most common dosage was 0.5 mg/kg/day (used in 6 studies) [1, 2, 10, 16, 22, 26]. Lower doses of 0.3 mg/kg/day were used in 3 studies [14, 15, 25]. The study comparing high dose (0.6 mg/kg/day) versus low dose (0.3 mg/kg/day) showed better clearance rate with high dose (76% vs 46%) [24]. The most common treatment period was 3 months (used in 8 studies) [1, 22–26, 28, 30]. Shorter durations of 1–2 months were used in 3 studies [2, 15, 16]. The longest treatment was 12 months [14]. In the case of efficacy, resolution rates ranged from 38% to 87.5%. The highest resolution rate (87.5%) was reported by Olguin Garcia et al. with 30 mg/day for 12 weeks [23]. Also, Dash et al. reported 87% resolution for verruca plana but only 61% for palmoplantar warts [26].

Follow-up periods ranged from 3 months to 3 years. Dave et al. reported the longest remission period (3 years) with a maintenance dose [30]. Rallis et al. reported relapse after 6 months, suggesting maintenance therapy may be necessary [14]. Cheilitis (dry lips) was the most commonly reported adverse event (5 studies). Dry skin was the second most common one (4 studies). One study reported laboratory abnormalities (elevated CPK, transferases, and cholesterol) [2]. Five studies did not mention adverse events at all [14, 15, 25, 28, 29].

Among the included studies, the most frequently used dosage was 0.5 mg/kg/day, reported in six studies [1, 2, 10, 26, 31, 32], followed by 0.3 mg/kg/day in three studies [14, 15, 24]. One study compared 0.6 mg/kg/day (high dose) and 0.3 mg/kg/day (low dose) [24], reporting a higher clearance rate with the high-dose group (76% vs. 46%). The most common treatment duration was 3 months, used in seven studies [1, 23, 24, 30, 32, 33]. Resolution rates varied from 38% to 87.5%, with the highest efficacy reported by Olguin Garcia et al. (87.5%) using a fixed dose of 30 mg/day for 12 weeks. Dash et al. also reported 87% clearance in verruca plana cases at 0.5 mg/kg/day over 3 months. These results suggest that three-month regimens of 0.5–0.6 mg/kg/day may offer the most favorable balance between efficacy and tolerability.

As far as secondary outcomes go, follow-up across the included studies ranged from 3 to 4 months in the trials and quasiexperimental studies to as long as 3 years in a maintenance case series [16, 19–23, 28]. Relapse after treatment cessation was documented, including recurrence around 6 months postdiscontinuation in the earliest report [14] and a 21% relapse within 4 months in a quasiexperimental cohort [16]. In the comparative study by Nooruldeen et al., recurrence was 13.3% with oral isotretinoin versus 31% with cryotherapy [19]. By contrast, maintenance low-dose isotretinoin (0.1–0.2 mg/kg/day) after induction was associated with prolonged remission of up to 3 years [28]. Formal patient-reported outcomes (e.g., satisfaction, cosmetic acceptability) were rarely collected and inconsistently reported across studies, precluding quantitative synthesis.

For quality assessment of all the studies, the JBI tool was used [21]. For case reports and case series, out of 8 studies, 6 were rated as “moderate” [1, 2, 27–30], 1 study was rated as low quality [15], and 1 study was rated as “good” quality [14] with the quality scores ranging from 4 to 7. Also, our only cross-sectional study was rated as “moderate” quality using the same quality assessment tool [30]. In two quasiexperimental studies which both were took place in Iraq, one of them was rated as “low” [16] and the other was rated as “moderate” [22] quality with the score of 3 and 5, respectively (out of 9). Finally, for the RCTs, 3 studies were rated as “moderate” [10, 24, 25] and 1 study was rated as “good” quality [23] with the score ranged from 7 to 12. All of the Indian and Egyptian studies have been classified as moderate quality in quality score assessment, while just two studies that were from Mexico and Greece classified as good quality. The details on the quality assessments of the manuscripts are shown in Tables 2, 3, 4, 5, and 6.

## 5. Discussion

Flat warts are a common therapeutic problem. Conventional therapies for HPV infection are often associated with unsatisfactory response rates and high recurrence rates [16]. There is no single treatment that is absolutely effective, and different types of treatment may be combined [30]. This review is the first to systematically evaluate oral isotretinoin for plane warts across diverse populations and study designs. A key strength is its comprehensive inclusion of both controlled trials and real-world clinical cases, supported by rigorous quality assessment using JBI tools. The most important finding is that oral isotretinoin, particularly at 0.5–0.6 mg/kg/day for 3 months, achieved clearance rates up to 87% with acceptable safety, highlighting its potential as an effective option for recalcitrant or widespread plane warts.

### 5.1. Therapeutic Effects

The efficacy of isotretinoin in treating plane warts has been well-documented across various studies, demonstrating significant clearance rates in different populations and anatomical regions. Studies such as Al-Hamamy et al. reported a 73% complete clearance rate for facial plane warts in adolescents and adults, with a low relapse rate (21%) over 4 months [16]. Similarly, Białecka et al. confirmed the effectiveness of isotretinoin for recalcitrant warts on the back of the hands, a site often resistant to conventional therapies [1]. Dash et al. provided further insights into its effectiveness across different wart subtypes, showing 87.09% clearance for verruca plana, whereas other wart types, such as genital warts, had lower response rates (30%) [26]. Therefore, regional variations in treatment response should be considered. While facial plane warts generally exhibit better response rates, potentially due to factors like local blood supply or HPV serotype distribution, isotretinoin's efficacy is not limited to facial lesions. For generalized lesions (e.g., Prateek et al., 2017; Bialecka et al., 2018; Nooruldeen et al., 2021), the effectiveness varied significantly [1, 22, 29]. While some studies reported complete resolution [1], others noted partial remission of the patients [10], indicating that generalized lesions might be more challenging to treat effectively.

While the exact mechanism through which isotretinoin exerts its therapeutic effect on HPV-infected cells remains not fully elucidated, current evidence suggests multiple pathways of action. The drug appears to modulate keratinocyte growth and differentiation, alter cellular cohesiveness, and inhibit HPV DNA replication within infected cells [24]. Researchers have observed an inverse correlation between retinoid concentration and HPV DNA levels in affected epithelial cells, suggesting a suppressive effect on viral replication [15]. Beyond these primary mechanisms, isotretinoin demonstrates immunomodulatory, anti-inflammatory, and proapoptotic properties that likely contribute to its therapeutic efficacy [1, 25]. Current investigations have further proposed that oral isotretinoin's antiangiogenic effects, specifically its ability to reduce vascular endothelial growth factor (VEGF) production by keratinocytes, may be particularly significant in treating warts dependent on angiogenesis [25, 30]. This multifaceted mechanism explains isotretinoin's effectiveness against various wart presentations, particularly those resistant to conventional treatments targeting only superficial manifestations of HPV infection.

Multiple studies by Nofal et al. [24, 25], Nooruldeen et al. [22], and Al-Hamamy et al. [16] have demonstrated oral isotretinoin's effectiveness across diverse age groups, including pediatric populations. This efficacy in younger patients may result from their more robust immune responses potentially facilitating faster viral clearance, higher skin regenerative capacity, and possibly different HPV genotype distributions compared to adults [10]. This finding has significant clinical implications, given the limited systemic treatment options available for children with extensive or treatment-resistant warts. However, these investigations did not specifically stratify or compare treatment outcomes or adverse event profiles across different age categories. Further research explicitly designed to examine age-related variations in treatment response through age-stratified analyses is necessary to better understand this clinically relevant factor. Such studies would provide valuable insights for optimizing isotretinoin protocols in diverse patient populations, particularly for pediatric patients, where treatment options may be more limited.

### 5.2. Dosage and Treatment Duration

The response to isotretinoin treatment appears to be dose-dependent. Higher dosages (0.6 mg/kg/day and above), as utilized in Nofal et al.'s high-dose group and Rallis et al.'s 0.8 mg/kg/day protocol, yield superior clearance rates but increase the likelihood of adverse effects, particularly mucocutaneous manifestations like cheilitis and xerosis [2, 14, 24]. Lower dosages (0.3 mg/kg/day and below) generally produce reduced clearance rates, exemplified by Nofal et al.'s low-dose group achieving only 46% clearance [24]. Notably, even minimal doses can achieve complete resolution in certain cases, particularly for facial lesions, as documented by Milijkovic et al. or in combination with other treatments such as Dave et al.'s maintenance approach using 0.1–0.2 mg/kg/day [15, 30]. Fixed dosing strategies, such as the 30 mg/day approach used by Olguin Garcia et al., which achieved 87.5% resolution rate, further complicate the picture, suggesting that weight-based dosing may not always be strictly necessary, particularly for facial plane warts where localized effectiveness is often pronounced [23]. These findings indicate that optimal dosing should be individualized, considering patient characteristics, lesion presentation, and treatment objectives while balancing efficacy against adverse effect profiles.

As suggested by some studies, doses below 0.5 mg/kg/day could be classified as a “low-dose” regimen, while doses exceeding 0.5 mg/kg/day might be categorized as a “high-dose” regimen of oral isotretinoin [24]. Also, as it was mentioned before, some researchers have employed fixed doses regardless of body weight, such as 20 mg/day [15], while others have utilized weight-based calculations, which further complicates the standardization of dosing protocols. Optimal dosing strategies for isotretinoin in plane warts remain a subject of ongoing investigation. The most common regimen across studies was 0.5 mg/kg/day [1, 2, 10, 16, 22, 26], which yielded substantial clearance rates with manageable side effects.

Lower dose isotretinoin regimens typically require extended treatment periods to achieve comparable cumulative doses, though they produce fewer adverse effects [2, 16]. For children with plane warts, the favorable safety profile of low-dose regimens may outweigh the disadvantage of prolonged treatment courses, particularly when considering the developmental concerns associated with higher isotretinoin exposures [16].

Treatment duration for oral isotretinoin typically ranges from 8 to 20 weeks. This duration is notably shorter than that required for many conventional therapies, including cryotherapy, which often necessitates treatment periods extending to 6 months [22]. The most common treatment period employed across the included studies, and often cited as optimal, is 3 months [10, 22–25, 28, 30]. Several investigations, including those by Dash et al., Nofal et al., and Nooruldeen et al., utilized a 3-month treatment duration and reported generally favorable outcomes, with resolution rates typically ranging from 69% to 87% [22, 25, 26]. This 3-month timeframe appears to strike a balance between achieving therapeutic efficacy and minimizing the cumulative risk of adverse events associated with prolonged isotretinoin exposure. However, the data also reveal nuances and complexities beyond a simple linear relationship between duration and effectiveness. Extending isotretinoin treatment beyond 3 months does not reliably produce better outcomes compared to standard 3-month protocols. This is evidenced by Rallis et al.'s 12-month study, which failed to prevent relapse and demonstrate superior results over shorter regimens [14].

Conversely, Milijkovic et al. and Olguin Garcia et al. both reported successful resolution with 1-month treatment durations in facial lesions, but the potential for relapse with such abbreviated courses warrants consideration [15, 23]. Furthermore, the concept of maintenance therapy strategies, involving a lower dose isotretinoin regimen administered after the initial high-dose treatment phase, adds another layer of complexity. Some studies suggest that incorporating a maintenance phase may be more crucial for achieving long-term remission and preventing recurrence than simply extending the initial high-dose treatment period [14, 30]. Dave et al., for example, reported the longest remission period of 3 years with a maintenance dose strategy, highlighting the potential benefits of extended, lower dose therapy in sustaining treatment response [30]. Interestingly, rapid response was observed in some studies regardless of planned duration, with Bialecka et al. reporting remission after just 3 weeks despite a planned 3-month course [1] and Olguin Garcia et al. noting resolution after just 1 month [2].

### 5.3. Comparison With Alternative Therapies

Isotretinoin has demonstrated superior efficacy compared to common wart treatments. Nooruldeen et al. (2021) compared isotretinoin to cryotherapy, which had a 49% cure rate with a 31% recurrence rate, whereas isotretinoin achieved an 85% improvement rate with significantly lower recurrence (13.3%) [22]. Similarly, Kaur et al. found oral isotretinoin to be more effective than topical isotretinoin, with 69% complete clearance vs. 38% for topical application [10]. This superiority is likely attributable to the systemic action of oral isotretinoin, offering better bioavailability and tissue penetration compared to topical formulations. This systemic approach is especially valuable for patients with extensive or multifocal lesions, where topical treatments would be impractical or ineffective.

In comparative analysis with intralesional Candida antigen, Nofal et al. found isotretinoin demonstrated slightly lower complete clearance rates (44.4% versus 55.6%) but exhibited a superior partial response rate (44.4%) compared to the Candida antigen group (11.1%) [25]. This response pattern suggests isotretinoin may serve a valuable role in combination therapeutic approaches targeting recalcitrant lesions. Furthermore, isotretinoin's systemic mechanism provides distinct advantages over destructive modalities, such as laser therapy, cryotherapy, and chemical peeling procedures [16, 22]. These benefits include reduced scarring formation, diminished inflammatory response, and minimized post-treatment pigmentary alterations [1, 25].

Given that plane warts readily koebnerize, destructive techniques like laser, cryotherapy, and chemical peeling might even be counterproductive by potentially exacerbating the condition [10]. Destructive methods are often associated with scarring, inflammation, and post-treatment pigmentation changes, which are less likely with systemic isotretinoin [1]. Isotretinoin's systemic nature addresses subclinical HPV infection, reducing recurrence and spread, which is especially beneficial for extensive or multifocal lesions where topical treatments are impractical or ineffective [16].

### 5.4. Safety, Tolerability, and Adverse Events

The most frequently reported adverse effects include cheilitis (up to 100%), dry skin (70%–80%), and dry nose (30%) [16, 23]. Dash et al. reported 78.7% cheilitis and 36.57% xerosis, but no serious systemic side effects [26]. Liver function and lipid profile abnormalities were observed in some studies, yet these remained within manageable limits and were reversible upon dose reduction [23].

While generally well-tolerated, isotretinoin is associated with dose-dependent side effects. Studies utilizing higher doses, such as Nofal et al.'s 0.6 mg/kg/day regimen approach, while reporting superior clearance rates, also noted a higher frequency of adverse events, particularly dry lips (cheilitis) and dry skin [24]. Al-Hamamy et al., while reporting good efficacy with the 0.5 mg/kg/day dosage, also acknowledged the prevalence of milder adverse events associated with this regimen [16]. In contrast, lower doses of isotretinoin, such as the 0.3 mg/kg/day regimen investigated by Nofal et al. and the even lower doses (0.1–0.2 mg/kg/day) used by Dave et al., were consistently associated with fewer reported adverse events [25, 30]. Nofal et al.'s low-dose group, while exhibiting a lower clearance rate (44.4%), also reported fewer adverse events compared to the high-dose group, suggesting that lower doses may be better tolerated, particularly for patients sensitive to side effects [25]. Dave et al.'s use of very low-dose isotretinoin (0.1–0.2 mg/kg/day) as a maintenance strategy, while still achieving complete resolution, further underscores the potential for lower doses to minimize adverse events while maintaining therapeutic benefit [30].

Treatment duration, in addition to dosage, also plays a significant role in influencing the incidence and severity of adverse events due to cumulative exposure. Dash et al. noted cheilitis (78.7%) and xerosis (61%) after 3 months [26], while Rallis et al. (2008) reported relapse and dryness 4 months after discontinuing an initial 6-month isotretinoin treatment [14]. Maintenance regimens mitigated this by using low doses postremission, reducing cumulative toxicity [30]. Notably, higher doses (0.6 mg/kg/day) of isotretinoin over a shorter period (up to 3 months) were associated with more frequent acute but transient side effects, whereas prolonged low-dose therapy has the potential to exacerbate chronic side effects such as dryness [14, 24].

Some studies did not mention any adverse events, which might reflect a lack of reported data or less aggressive treatment protocols [14, 15, 25, 28, 29]. More serious adverse events associated with isotretinoin, such as pancreatitis, inflammatory bowel disease, and idiopathic intracranial hypertension, were not observed in the reviewed clinical trials, possibly due to limited sample sizes and relatively short follow-up periods. Other adverse effects, including psychiatric symptoms and idiopathic intracranial hypertension, remain controversial. While some studies reported a potential link to depression, larger scale reviews failed to confirm a significant association [22]. Importantly, teratogenicity remains a critical concern, requiring stringent contraceptive measures for female patients of reproductive age [1, 23, 24].

### 5.5. Therapeutic Application in Combination Regimens

Combination therapy has emerged as a promising approach to enhance the efficacy of isotretinoin while mitigating its limitations. The rationale for combination therapy stems from the observation that monotherapies for plane warts are often associated with treatment resistance and high recurrence potential [1]. By targeting multiple pathophysiological mechanisms simultaneously, combination approaches may overcome these limitations and achieve superior outcomes. Nofal et al. compared isotretinoin monotherapy with intralesional Candida antigen, finding that while Candida antigen alone had the highest complete clearance rate (55.6%), the combination therapy resulted in the lowest nonresponse rate (5.6%) [25]. This approach leverages both the antiviral and keratinocyte-modulating properties of isotretinoin and the immunostimulatory effects of Candida antigen. This suggests that combining isotretinoin with immune-modulating agents may optimize wart clearance rates.

Another study by Dave et al. explored low-dose isotretinoin (0.1–0.2 mg/kg/day) in combination with topical treatments (5-fluorouracil, imiquimod), showing 100% clearance with prolonged remission (up to 3 years) [30]. This aligns with the broader dermatologic understanding that systemic retinoids may enhance the effects of topical keratolytic agents and immune response modulators. Furthermore, combination approaches may be especially valuable for recalcitrant cases that have failed to respond to multiple previous interventions [15, 30]. Despite these promising observations, the optimal combination regimens, dosing schedules, and treatment durations remain to be established through well-designed comparative trials. Future research should focus on identifying specific patient populations most likely to benefit from combination approaches and establishing standardized protocols to guide clinical practice.

## 6. Limitations

This study has several important limitations that should be recognized. By only including English publications, we may have missed valuable research in other languages. The analysis is also constrained by the small number of available studies and their limited sample sizes. The fact that most included studies came from Egypt and India raises questions about how applicable these findings are to populations with different genetic profiles, environmental factors, and HPV strain distributions. Additionally, the included studies generally had methodological weaknesses, lacking randomization and triple-blinding, which introduces potential biases and reduces confidence in the results. Most included studies were rated moderate-to-low quality on JBI tools, with only a minority achieving “good” ratings. Accordingly, the certainty of evidence for both efficacy and safety is low, and all effect estimates should be interpreted cautiously. Some included papers did not report anything on the adverse events, making us cautious about underestimating. Future research should address these issues through randomized, triple-blind experimental designs with much larger participant groups. These improvements would significantly enhance the quality of evidence by better controlling for confounding variables, reducing bias risk, and increasing statistical power. This would create a stronger foundation for clinical decisions about using isotretinoin to treat plane warts across diverse patient populations and healthcare settings.

## 7. Conclusion

This systematic review highlights oral isotretinoin as a potentially effective and well-tolerated treatment for plane warts. The reviewed studies demonstrated clearance rates of up to 87%, especially in recalcitrant or facial lesions, with cheilitis and dry skin being the most common adverse effects. While the evidence is promising, limitations such as small sample sizes and moderate methodological quality make us cautious in a wide interpretation. Further high-quality randomized trials are needed to establish standardized dosing protocols and confirm long-term safety and efficacy.

## Figures and Tables

**Table 1:** The characteristics of the included studies.

	Author and year	Sample size (female/male)/age	Lesion site	Intervention/dosage/period of treatment	Outcome/adverse events	Ref
1	Rallis et al. 2008	1 (male)/20y	Face/extremities	Oral isotretinoin 0.8 mg/kg/day for 12 months—followed by a maintenance dose of 0.3 mg/kg/day (20 mg/day)	Relapse after 6 months and remission at last/no adverse events mentioned	[13]

2	Milijkovic et al. 2012	2 (40 y male/21 y female)	Face	20 mg daily (0.3 mg/kg/day) oral isotretinoin for 1 month	Resolution of lesions after a month and no relapse after 6 months/no adverse events mentioned	[14]
			Face	20 mg daily (0.4 mg/kg/day) oral isotretinoin for 1 month	Resolution of lesions after a month and no relapse after 12 months/no adverse events mentioned	

3	Al-Hamamy et al. 2012	26 patients/15.28 ± 8.51 years	Face	0.5 mg/kg/day for 2 months	78.9% resolution rate after 4 months of follow-up/dry lips, dry skin	[15]

4	Olguin Garcia et al. 2015	16 patients (13 f/3 m) in isotretinoin and 15 cases (8 f/7 m) in placebo group/median age of 25 in isotretinoin group and 21 in placebo group	—	30 mg/day for 12 weeks	87.5% resolution rate in the treatment group/cheilitis, pruritus, dry skin, and dry eyes	[19]

5	Kaur et al. 2017	16 patients of Group A and 13 patients of Group B/27.84 ± 6.98 in Group A and 31.56 ± 11.92 in Group B	Face/trunk/extremities	A: 0.5 mg/kg/day oral isotretinoin	A: 69% complete remission, 31% partial remission	[20]
				B: topical isotretinoin gel for 3 months	B: 38% complete remission 46% partial remission/cheilitis	

6	Prateek et al. 2017	1 (f)/13 y	Generalized	Oral isotretinoin	Not mentioned/no adverse events mentioned	[24]

7	Dash et al. 2018	82 cases of verrucae plana and palmoplantar warts/most of the patients between 21 and 30	—	0.5 mg/kg/day of isotretinoin for 3 months	87% of verruca plana cases and 61% of palmoplantar cases had complete resolution/cheilitis, xerosis, hair loss	[30]

8	Bialecka et al. 2018	1 (f)/31 y	Upper extremity	0.5 mg/kg daily for 3 months	Remission after 3 weeks/dry lips and dryness of conjunctiva	[25]

9	Ozturk et al. 2018	1 (f)/16 y	Face and neck	Isotretinoin 30 mg/day for 3 months	Near total resolution of lesions after 3 months/no adverse events mentioned	[31]

10	Girijala et al. 2019	1 (m)/27 y	Generalized	Only responsive to systemic isotretinoin when the patient was aged 9–12 years old		[27]

11	Dave et al. 2019	14 (4f/10 m)	Generalized	0.1–0.2 mg/kg/day isotretinoin for 3-month course with a maintenance dose	Complete resolution of warts and a remission period of 3 years/no adverse events mentioned	[29]

12	Nofal et al. 2020	108 patients in 3 groups (48 m and 60 f)/10.67 ± 7.24 in Group 1/15.39 ± 11.28 in Group 2/15.22 ± 9.28 in Group 3	—	Group 1: 0.3 mg/kg/day oral isotretinoin for 3 months. Group 2: intralesional injection of Candida antigen Group 3: combination	44.4% clearance of lesions in the oral isotretinoin alone group, 55.6% in the Candida group, and 38.8% in the combination group/no adverse events mentioned	[32]

13	Nooruldeen et al. 2021	40 patients (67.5% f and 32.5% m)/mean age of 18	—	0.5 mg/kg/day in two divided doses for 3 months	85% effectiveness after 3 months follow-up/cheilitis, headache, low mood, dry skin	[33]

14	Nofal et al. 2022	100 patients in 2 groups (22f and 28 m in Group 1 and 19 f and 31 m in Group 2)/19.3 ± 8.65 in Group 1 and 23.8 ± 9.2 in Group 2		Group 1: 0.6 mg/kg/day (high-dose isotretinoin) Group 2: 0.3 mg/kg/day (low-dose isotretinoin) For a maximum of 3 months	76% clearance rate in high-dose group and 46% in the low-dose isotretinoin group/recurrence was higher in low-dose group/cheilitis, dry skin	[21]

15	Olguin Garcia et al. 2023	1 (m)/27 y	Face	Started the treatment with 0.5 mg/kg/day oral isotretinoin for a month and after that due to a rise in CPK, transferases, and cholesterol, the dose was cut in half and finished a 3-month course	Resolution of the lesion after a month/laboratory changes	[28]

**Table 2:** Methodological quality assessment for case report included studies using the Joanna Briggs Institute (JBI) tool.

First author, year	Q1	Q2	Q3	Q4	Q5	Q6	Q7	Q8	Final score	Quality
Rallis 2008	Y	Y	Y	Y	Y	Y	N	Y	7	Good
Prateek 2017	Y	Y	Y	Y	N	N	N	Y	5	Moderate
Bialecka 2018	Y	Y	Y	N	N	Y	Y	Y	6	Moderate
Ozturk 2018	Y	N	Y	N	N	Y	N	Y	4	Moderate
Girijala 2019	Y	Y	Y	Y	N	N	N	Y	5	Moderate
Olguin Garcia 2023	Y	N	Y	N	Y	Y	Y	Y	6	Moderate

*Note:* Y: Yes, N: No. Q1. Were the patient's demographic characteristics clearly described? Q2. Was the patient's history clearly described and presented as a timeline? Q3. Was the current clinical condition of the patient on presentation clearly described? Q4. Were diagnostic tests or methods and the results clearly described? Q5. Were the intervention(s) or treatment procedure(s) clearly described? Q6. Was the postintervention clinical condition clearly described? Q7. Were adverse events (harms) or unanticipated events identified and described? Q8. Does the case report provide takeaway lessons? Rating—Good, Medium, or Poor; Good = (≥ 7 yes), Moderate = (4–6), and Low = (≤ 3 yes).

**Table 3:** Methodological quality assessment for case series included studies using the Joanna Briggs Institute (JBI) tool.

First author, year	Q1	Q2	Q3	Q4	Q5	Q6	Q7	Q8	Q9	Q10	Final score	Quality
Milijkovic 2012	N	N	N	U	U	Y	Y	Y	N	NA	3	Low
Dave 2019	Y	N	N	U	U	Y	Y	Y	Y	NA	5	Moderate

*Note:* Y: Yes, N: No, U: Unclear. Q1. Were there clear criteria for inclusion in the case series? Q2. Was the condition measured in a standard, reliable way for all participants included in the case series? Q3. Were valid methods used for the identification of the condition for all participants included in the case series? Q4. Did the case series have consecutive inclusion of participants? Q5. Did the case series have complete inclusion of participants? Q6. Was there clear reporting of the demographics of the participants in the study? Q7. Was there clear reporting of clinical information of the participants? Q8. Were the outcomes or follow-up results of cases clearly reported? Q9. Was there clear reporting of the presenting sites'/clinics' demographic information? Q10. Was statistical analysis appropriate? Rating—Good, Medium, or Poor; Good = (≥ 7 yes), Moderate = (4–6), and Low = (≤ 3 yes).

Abbreviation: NA, not applicable.

**Table 4:** Methodological quality assessment for analytical cross-sectional included studies using the Joanna Briggs Institute (JBI) tool.

First author, year	Q1	Q2	Q3	Q4	Q5	Q6	Q7	Q8	Final score	Quality
Dash 2018	Y	Y	Y	N	N	N	Y	Y	5	Moderate

*Note:* Y: Yes, N: No. Q1. Were the criteria for inclusion in the sample clearly defined? Q2. Were the study subjects and the setting described in detail? Q3. Was the exposure measured in a valid and reliable way? Q4. Were objective, standard criteria used for measurement of the condition? Q5. Were confounding factors identified? Q6. Were strategies to deal with confounding factors stated? Q7. Were the outcomes measured in a valid and reliable way? Q8. Was appropriate statistical analysis used? Rating—Good, Medium, or Poor; Good = (≥ 7 yes), Moderate = (4–6), and Low = (≤ 3 yes).

**Table 5:** Methodological quality assessment for quasiexperimental included studies using the Joanna Briggs Institute (JBI) tool.

First author, year	Q1	Q2	Q3	Q4	Q5	Q6	Q7	Q8	Q9	Final score	Quality
Al-Hamamy 2012	Y	N	NA	NA	N	U	U	Y	Y	3	Low
Nooruldeen 2021	Y	N	NA	NA	Y	Y	U	Y	Y	5	Moderate

*Note:* Y: Yes, N: No, U: Unclear. Q1. Is it clear in the study what is the “cause” and what is the “effect” (i.e., there is no confusion about which variable comes first)? Q2. Was there a control group? Q3. Were the participants included in any comparisons similar? Q4. Were the participants included in any comparisons receiving similar treatment/care, other than the exposure or intervention of interest? Q5. Were there multiple measurements of the outcome both pre and post the intervention/exposure? Q6. Were the outcomes of participants included in any comparisons measured in the same way? Q7. Were outcomes measured in a reliable way? Q8. Was follow-up complete and if not, were differences between groups in terms of their follow-up adequately described and analyzed Q9. Was appropriate statistical analysis used? Rating—Good, Medium, or Poor; Good = (≥ 7 yes), Moderate = (4–6), and Low = (≤ 3 yes).

Abbreviation: NA, not applicable.

**Table 6:** Methodological quality assessment for randomized controlled trials' included studies using the Joanna Briggs Institute (JBI) tool.

First author, year	Q1	Q2	Q3	Q4	Q5	Q6	Q7	Q8	Q9	Q10	Q11	Q12	Q13	Final score	Quality
Olguin Garcia 2015	Y	Y	Y	Y	Y	Y	Y	Y	U	Y	Y	Y	Y	12	Good
Kaur 2017	Y	Y	U	NA	NA	Y	NA	U	U	Y	Y	Y	Y	7	Moderate
Nofal 2020	Y	Y	Y	NA	NA	Y	NA	U	U	Y	Y	Y	Y	8	Moderate
Nofal 2022	Y	Y	Y	U	U	Y	U	U	U	Y	Y	Y	Y	8	Moderate

*Note:* Y: Yes, U: Unclear. Q1. Was true randomization used for assignment of participants to treatment groups? Q2. Was allocation to treatment groups concealed? Q3. Were treatment groups similar at the baseline? Q4. Were participants blind to treatment assignment? Q5. Were those delivering treatment blind to treatment assignment? Q6. Were treatment groups treated identically other than the intervention of interest? Q7. Were outcomes assessors blind to treatment assignment? Q8. Were outcomes measured in the same way for treatment groups? Q9. Were outcomes measured in a reliable way? Q10. Was follow-up complete and if not, were differences between groups in terms of their follow-up adequately described and analyzed? Q11. Were participants analyzed in the groups to which they were randomized? Q12. Was appropriate statistical analysis used? Q13. Was the trial design appropriate, and any deviations from the standard RCT design (individual randomization, parallel groups) accounted for in the conduct and analysis of the trial? Rating—Good, Medium, or Poor; Good = (≥ 10 yes), Moderate = (6–9 yes), and Low = (≤ 5 yes).

Abbreviation: NA, not applicable.

## Supporting Information

Supporting Table S1: The details of search strategy.
