# Supplementary material for: Efficacy and Safety of Oral Isotretinoin in Plane Warts: A Systematic Review on Clinical Studies
Source: Scientifica (Cairo). 2025 Oct 22;2025:4242268. doi: 10.1155/sci5/4242268 (PMC12571534; doi:10.1155/sci5/4242268)
Supplement: Supporting Information — Additional supporting information can be found online in the Supporting Information section. [file 4242268.f1.docx]

Table s1. The details of search strategy

| Results | Search string | Database |
| --- | --- | --- |
| 21 | (("isotretinoin"[MeSH Terms] OR "isotretinoin"[All Fields] OR "13 cis retinoic acid"[All Fields] OR "13-cis-retinoic acid"[All Fields] OR "roaccutane"[All Fields] OR "accutane"[All Fields] OR "absorica"[All Fields] OR "myorisan"[All Fields] OR "claravis"[All Fields] OR "amnesteem"[All Fields] OR "isotretinoin zinc salt"[All Fields] OR "ro 4 3780"[All Fields] OR "ro 43780"[All Fields] OR "isotretinoinum"[All Fields] OR "tretinoin"[All Fields]) AND ("Warts"[MeSH Terms] OR "wart"[All Fields] OR "warts"[All Fields] OR "verruca"[All Fields] OR "verrucae"[All Fields] OR "viral warts"[All Fields] OR "common warts"[All Fields]) AND ("plane"[All Fields] OR "plana"[All Fields] OR "planae"[All Fields] OR "flat"[All Fields] OR "Juvenile"[All Fields] OR "planar"[All Fields] OR "verruca plana"[All Fields] OR "verrucae planae"[All Fields])) | PubMed |
|  | [Link](https://pubmed.ncbi.nlm.nih.gov/?term=%28%28%22isotretinoin%22%5BMeSH+Terms%5D+OR+%22isotretinoin%22%5BAll+Fields%5D+OR+%2213+cis+retinoic+acid%22%5BAll+Fields%5D+OR+%2213-cis-retinoic+acid%22%5BAll+Fields%5D+OR+%22roaccutane%22%5BAll+Fields%5D+OR+%22accutane%22%5BAll+Fields%5D+OR+%22absorica%22%5BAll+Fields%5D+OR+%22myorisan%22%5BAll+Fields%5D+OR+%22claravis%22%5BAll+Fields%5D+OR+%22amnesteem%22%5BAll+Fields%5D+OR+%22isotretinoin+zinc+salt%22%5BAll+Fields%5D+OR+%22ro+4+3780%22%5BAll+Fields%5D+OR+%22ro+43780%22%5BAll+Fields%5D+OR+%22isotretinoinum%22%5BAll+Fields%5D+OR+%22tretinoin%22%5BAll+Fields%5D%29%0D%0AAND%0D%0A%28%22Warts%22%5BMeSH+Terms%5D+OR+%22wart%22%5BAll+Fields%5D+OR+%22warts%22%5BAll+Fields%5D+OR+%22verruca%22%5BAll+Fields%5D+OR+%22verrucae%22%5BAll+Fields%5D+OR+%22viral+warts%22%5BAll+Fields%5D+OR+%22common+warts%22%5BAll+Fields%5D%29%0D%0AAND%0D%0A%28%22plane%22%5BAll+Fields%5D+OR+%22plana%22%5BAll+Fields%5D+OR+%22planae%22%5BAll+Fields%5D+OR+%22flat%22%5BAll+Fields%5D+OR+%22Juvenile%22%5BAll+Fields%5D+OR+%22planar%22%5BAll+Fields%5D+OR+%22verruca+plana%22%5BAll+Fields%5D+OR+%22verrucae+planae%22%5BAll+Fields%5D%29%29%0D%0A&sort=date) |  |
| 60 | TITLE-ABS-KEY ((isotretinoin OR "13-cis-retinoic acid" OR "13 cis retinoic acid" OR roaccutane OR accutane OR absorica OR myorisan OR claravis OR amnesteem OR "isotretinoin zinc salt" OR "ro 4 3780" OR "ro 43780" OR isotretinoinum OR tretinoin) AND (wart OR warts OR verruca OR verrucae OR "viral warts" OR "common warts") AND (plane OR plana OR planae OR flat OR juvenile OR planar OR "verruca plana" OR "verrucae planae")) | Scopus |
|  | [Link](https://www.scopus.com/results/results.uri?sort=plf-f&src=s&sid=e2ff427d27065e7be19760d51cf83796&sot=a&sdt=a&sl=423&s=TITLE-ABS-KEY%28%28isotretinoin+OR+%2213-cis-retinoic+acid%22+OR+%2213+cis+retinoic+acid%22+OR+roaccutane+OR+accutane+OR+absorica+OR+myorisan+OR+claravis+OR+amnesteem+OR+%22isotretinoin+zinc+salt%22+OR+%22ro+4+3780%22+OR+%22ro+43780%22+OR+isotretinoinum+OR+tretinoin%29+AND+%28wart+OR+warts+OR+verruca+OR+verrucae+OR+%22viral+warts%22+OR+%22common+warts%22%29+AND+%28plane+OR+plana+OR+planae+OR+flat+OR+juvenile+OR+planar+OR+%22verruca+plana%22+OR+%22verrucae+planae%22%29%29&origin=searchadvanced&editSaveSearch=&txGid=83e55e598e762e51c4c2018962cf492a&sessionSearchId=e2ff427d27065e7be19760d51cf83796&limit=10) |  |
| 36 | ('isotretinoin'/exp OR '13 cis retinoic acid' OR 'roaccutane' OR 'accutane' OR 'isotretinoin zinc salt' OR 'ro 4 3780' OR 'ro 43780' OR 'absorica' OR 'myorisan' OR 'claravis' OR 'amnesteem') AND ('wart'/exp OR 'verruca'/exp OR 'verruca' OR 'warts') AND ('plane' OR 'plana' OR 'planae' OR 'flat' OR 'juvenile') | Embase |
|  | [Link](https://www.embase.com/#advancedSearch/resultspage/history.1/page.1/50.items/orderby.date/source.) |  |

**Date of search:** November 14, 2024
